# Effect of Yi Guan Jian decoction on differentiation of bone marrow mesenchymal stem cells into hepatocyte-like cells in dimethylnitrosamine-induced liver cirrhosis in mice

**DOI:** 10.3892/mmr.2021.12448

**Published:** 2021-09-17

**Authors:** Yan Xiang, Bing-Yao Pang, Yuan Zhang, Qiao-Ling Xie, Ying Zhu, Ai-Jing Leng, Long-Qing Lu, Hai-Long Chen

Mol Med Rep 15: 613-626, 2017; DOI: 10.3892/mmr.2016.6083

Following the publication of the above article, an interested reader to the authors’ attention that there appeared to be several duplications of data panels featured within Fig. 1,Fig. 2,Fig. 3. After having consulted their original data, the authors have realized that a number of the data panels were inadvertently assembled incorrectly in these figures.

The corrected versions of Fig. 1A (showing the correct data for the NC-2W and NC-4W experiments), Fig. 1B (including the correct data for the C-4W, M-2W, NC-2W and NC-4W experiments), Fig. 2 (showing the correct data for the YGD-2W experiment), Fig. 3A (NC-3W data panel corrected), Fig. 3B (HGF-1W and NC-3W data panels corrected) and Fig. 3C (C-4W data panel corrected) are shown on the next four pages. All these corrections were approved by all authors. The authors regret that these errors were not resolved before the publication of the paper, thank the Editor of *Molecular Medicine Reports* for granting them the opportunity to publish this corrigendum, and apologize to the readership for any inconvenience caused.

## Figures and Tables

**Figure 1.**
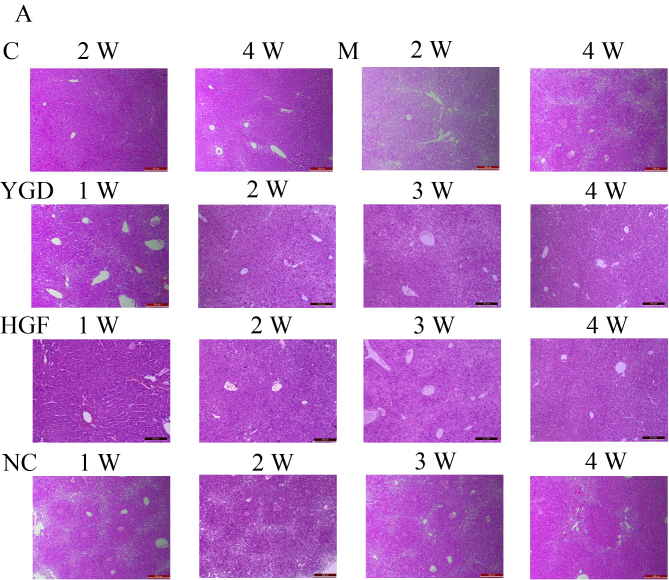

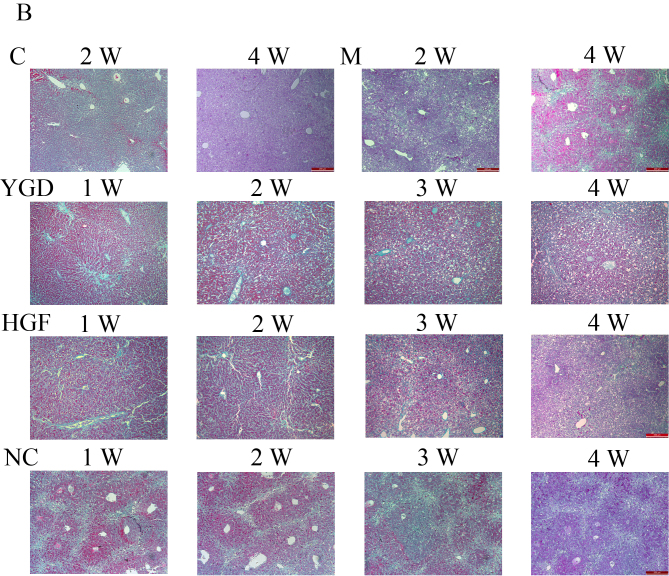
Comparison of liver histology in mice with DMN-induced liver cirrhosis. Mice were injected with DMN or normal saline for 4 weeks. Following 4 weeks of DMN treatment, mice were subsequently treated with YGD or HGF for a further 4 weeks. Liver tissues were stained with (A) hematoxylin and eosin and (B) Masson's trichrome. Liver damage induced by DMN was alleviated by YGD or HGF treatment. Magnification, ×100; scale bar=200 µm. YGD, Yi Guan Jian decoction; HGF, hepatocyte growth factor; DMN, dimethylnitrosamine; C, control; M, model; NC, negative control; W, week.

**Figure 2.**
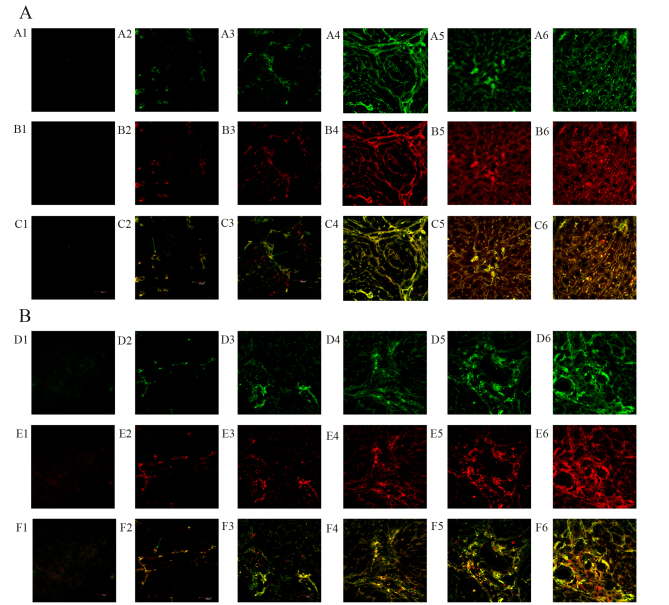
DMN and YGD regulate the differentiation of BMSCs into hepatocytes and biliary epithelial cells. Mice were injected with DMN or normal saline for 4 weeks. Following 4 weeks of DMN treatment, mice were treated with YGD or HGF for a further 4 weeks. (A) The expression of CD90 (green, top row) and Alb (red, middle row) was detected by immunofluorescence, with the co-localization analyzed by merging these images (bottom row). (B) The expression of CD90 (green, top row) and CK18 (red, middle row) was detected by immunofluorescence, with the co-localization analyzed by merging these images (bottom row). Expression and co-localization was increased by DMN injection and further increased by YGD treatment. Magnification, ×60. YGD, Yi Guan Jian decoction; HGF, hepatocyte growth factor; DMN, dimethylnitrosamine; CD, cluster of differentiation; Alb, Albumin; CK18, cytokeratin 18; C, control; M, model; W, week.

**Figure 3.**
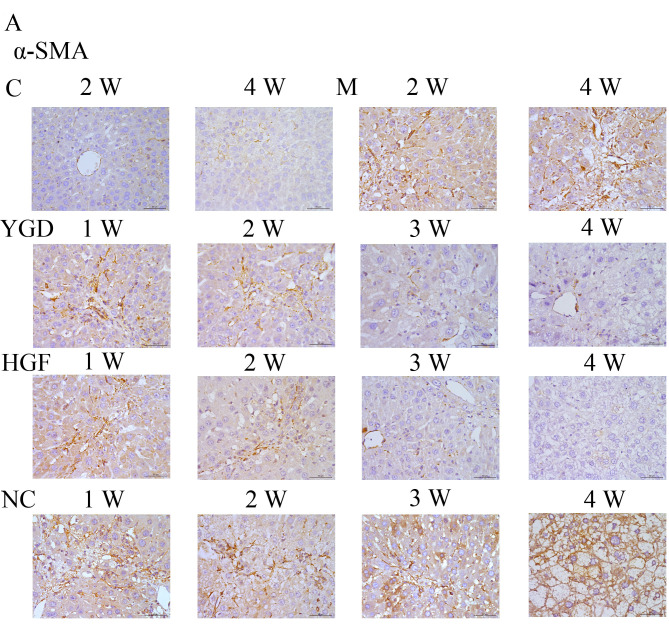

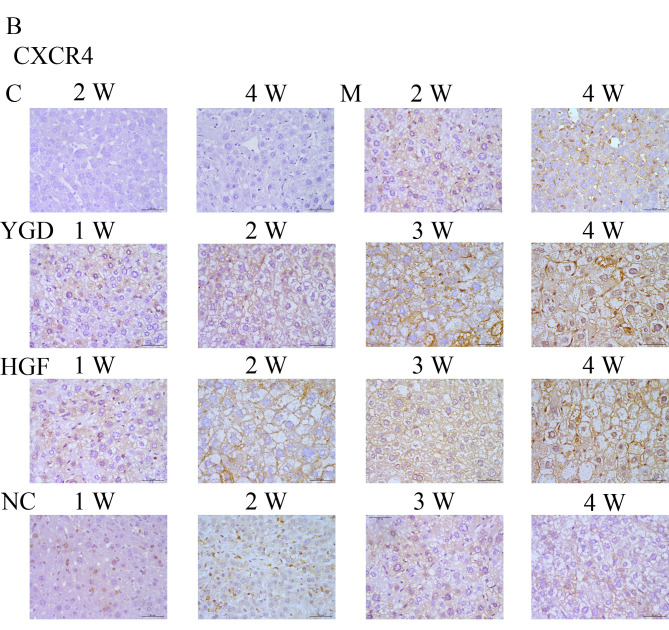

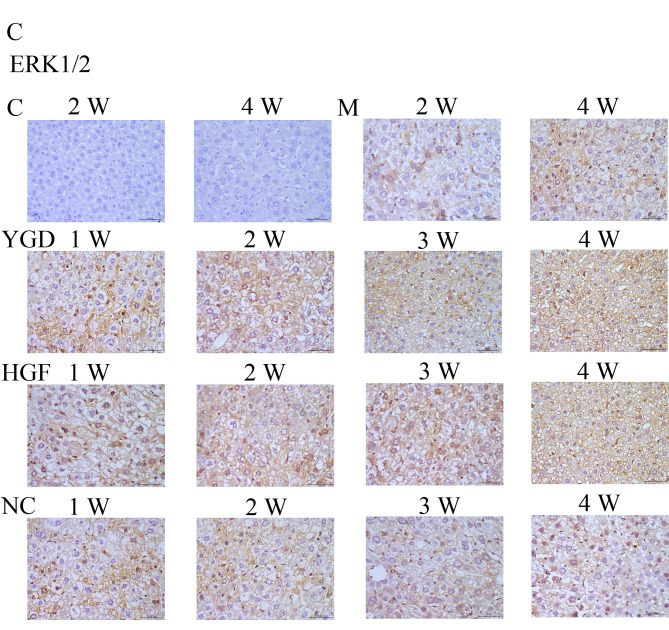
DMN, YGD and HGF regulate the expression of α-SMA, CXCR4, ERK1/2, NF-κB p65 and β-catenin. Mice were injected with DMN or normal saline for 4 weeks. Following 4 weeks of DMN treatment, mice were treated with YGD or HGF for a further 4 weeks. Liver sections were stained for (A) α-SMA (B) CXCR4. (C) ERK1/2.

